# Prenatal Methyl-Donor Supplementation Augments Colitis in Young Adult Mice

**DOI:** 10.1371/journal.pone.0073162

**Published:** 2013-08-19

**Authors:** Sabina A. Mir, Dorottya Nagy-Szakal, Scot E. Dowd, Reka G. Szigeti, C. Wayne Smith, Richard Kellermayer

**Affiliations:** 1 Section of Pediatric Gastroenterology, Hepatology and Nutrition, Department of Pediatrics, Baylor College of Medicine, Texas Children’s Hospital, Department of Agriculture/ARS Children’s Nutrition Research Center, Houston, Texas, United States of America; 2 MR DNA (Molecular Research), Shallowater, Texas, United States of America; 3 Department of Pathology, Baylor College of Medicine, Houston, Texas, United States of America; 4 Section of Infectious Disease, Department of Pediatrics, Baylor College of Medicine, Department of Agriculture/ARS Children’s Nutrition Research Center, Houston, Texas, United States of America; Virginia Tech, United States of America

## Abstract

Inflammatory bowel diseases (IBD) have become highly prevalent in developed countries. Environmentally triggered exaggerated immune responses against the intestinal microbiome are thought to mediate the disorders. The potential dietary origins of the disease group have been implicated. However, the effects of environmental influences on prenatal developmental programming in respect to orchestrating postnatal microbiome composition and predilection towards mammalian colitis have not been examined. We tested how transient prenatal exposure to methyl donor micronutrient (MD) supplemented diets may impact predilection towards IBD in a murine dextran sulfate sodium (DSS) colitis model. Prenatal MD supplementation was sufficient to modulate colonic mucosal *Ppara* expression (3.2 fold increase; p=0.022) and worsen DSS colitis in young adulthood. The prenatal dietary exposure shifted the postnatal colonic mucosal and cecal content microbiomes. Transfer of the gut microbiome from prenatally MD supplemented young adult animals into germ free mice resulted in increased colitis susceptibility in the recipients compared to controls. Therefore, the prenatal dietary intervention induced the postnatal nurturing of a colitogenic microbiome. Our results show that prenatal nutritional programming can modulate the mammalian host to harbor a colitogenic microbiome. These findings may be relevant for the nutritional developmental origins of IBD.

## Introduction

Inflammatory bowel diseases (IBD) compromising ulcerative colitis (UC) and Crohn disease (CD) are an emerging global healthcare problem [1]. Epidemiological studies report a continually rising incidence of the disorders not only in developed countries [2], but in developing parts of the world, such as Asia as well [3]. The etiology of IBD is unknown, but it appears to involve an exaggerated immune response against the gut microbiome in genetically susceptible individuals triggered by environmental factors [4,5]. However, the rising incidence and the high monozygotic twin discordant rates [6] of IBD cannot be explained by genetic predisposition. Nutritional and environmental characteristics of the westernized lifestyle are thought to be at least in part responsible for the increasing prevalence of IBD [7]. The developmental origins hypothesis proposes that during critical periods of mammalian development, environmental stimuli, including nutrition, can influence developmental pathways and induce permanent changes in metabolism and disease susceptibility. This hypothesis may be pertinent to the pathogenesis of IBD [8,9].

One group of molecular mechanisms, which has been recognized to dynamically respond to environmental influences, is designated as epigenetic. These processes may be involved in the developmental origins of chronic diseases [10,11]. Epigenetic modifications are mitotically heritable molecular changes that can modify gene expression without alterations in the genetic code. These processes can contribute to phenotypic changes in mammals allowing for dynamic adjustments to environmental stimuli [12]. The most stable epigenetic modification is the methylation of DNA cytosines at CpG dinucleotides, which is catalyzed by DNA methyltransferases. DNA methyltransferases utilize the mammalian one carbon pool, which can respond to nutritional modifications [13]. Maternal dietary supplementation of methyl-donor (MD) micronutrients (B12, folate, betaine and choline) has been found to be effective in altering the developmental establishment of DNA methylation at select murine genomic loci and to correlate with phenotype modifications [14]. All of these compounds can be found in various prenatal vitamins and supplements. The consumption of these micronutrients has become common in the developed world during pregnancy [15,16]. Chronic supplementation of folate and the maternal supplementation of numerous micronutrients have raised questions in regards to their potential role in the developmental origins of common human disorders with persistently rising incidence, such as asthma, autism [17,18] and IBD [19].

We have shown that maternal supplementation with MDs during pregnancy and lactation lead to increased acute colitis susceptibility in murine offspring [19]. This phenotype modification was associated with persistent DNA methylation and gene expression changes in addition to a prolonged separation of the colonic mucosal microbiomes. However, the effects of the individual MD micronutrients in this experimental model were not tested. The critical developmental period (prenatal versus postnatal) for the MDs to exert their colitis modifying effect was not determined either. Additionally, the select role of the transient MD supplementation induced microbiome separation was not interrogated. In thus study, we set forth to examine these questions.

## Materials and Methods

### Animals and diets

C57BL/6J (Jackson Laboratories, Bar Harbor, ME, USA) and germ free male Swiss-Webster (Taconic Farms Inc. Hudson, NY, USA) mice were used in the study. All experimental animals were maintained in accordance with the National Institutes of Health (NIH) Guide for the Care and Use of Laboratory animals, and the experiments were approved by the Institutional Review Board of the Baylor College of Medicine.

NIH-31 control diet (C) (TD #95262, Harlan-Teklad, Madison, WI, USA), or a methyl-donor (MD) supplemented diet (NIH-31 supplemented to 5 mg/Kg folic acid [2.5x control]; 0.5 mg/Kg vitamin B12 [~8x control]; 5 g/Kg betaine [not determined in control]; and 5.76 g/Kg choline [~3x control] [TD#01308, Harlan-Teklad, Madison, WI, USA]) [20] was used.

### Dietary Composition Effects

We investigated the influence of each component (folic acid [FA], betaine, vitamin B12 [B12], and choline) of the methyl-donor (MD) diet on offspring colitis susceptibility. Twelve C57BL/6J females 10 week old were used. Two females (for each group) were randomly assigned to either NIH-31 control diet (C) or to a MD supplemented diet, or to a diet with only one of the MD components supplemented, respectively for 2 weeks prior to mating, throughout gestation and lactation. At 12 weeks of age the females were housed with age matched C57BL/6J males. A total of 12 litters were generated from 6 different maternal dietary groups. The experiment was performed in two independent replicates. Total number of pups from control were 20, MD n= 20, betaine n=19, folic acid n=25 and choline n= 16. At postnatal days 21 (P21), the pups were weaned to the control diet until P90 (69 day reversal). Consequently, only persistent effects of maternal MD, or single nutrient supplementation were tested. At P90, chemical colitis was induced with DSS (described below). Body weight and signs of colitis/distress (diarrhea, hematochezia, pilorection of fur) were monitored daily during and after the DSS exposure. On day 10 (when weight loss usually peaks after 5 days of DSS exposure) mice were euthanized by CO_2_ asphyxiation. Mice that demonstrated excess weight loss (more than 25%), or signs of significant distress were euthanatized earlier (mortality group). At the end of the experiment, colonic lengths were measured. Histological severity of inflammation was determined by a blinded pathologist based upon a colitis scoring system modified from Albert, et al. [21,22].

### Cross-fostering experiments

We determined the critical period of development (*in utero* versus postnatal) during which MD supplementation may increase colitis susceptibly in young adulthood. In order to do so, we conducted a cross-fostering experiment. Eight virgin C57BL/6J females, 10 weeks of age, were assigned to control diet and 8 other females to the MD supplemented diet. The respective diets were provided to the females for 2 weeks prior to mating, during pregnancy and lactation (see above). Within 48 hours of delivery, each litter was crossed fostered to either a dam on the same diet (MD-MD as positive control and C-C as negative control), or to a dam on a different diet (MD-C or C-MD). There were 2 different litters within each cross fostered group (Figure S1). No pups were lactated by their biological dam. At P21, the pups were weaned to the control (NIH-31) diet until P90 (69 day reversal). At P90, 5 mice from 2 different cages (to eliminate cage bias) in the MD-MD and C-C groups, and 7 from MD-C and C-MD groups were euthanized by CO2 asphyxiation without DSS exposure for molecular analysis purposes. Feces from the cecum and colonic mucosal scrapings were collected for microbiome analysis and fecal transfer experiments (described below). The remaining mice were given 3% DSS in drinking water *ad libitum* for 5 days, followed by regular water exposure for an additional 5 days. The mice were weighed daily during this period. At day 10 following DSS exposure, the mice were euthanized by CO2 asphyxiation. Colonic length was measured and histological severity of inflammation was determined as described below.

### Dextran sulfate sodium (DSS) colitis

Susceptibility to colitis for C57BL/6J mice was tested by administering 3% (wt/vol) dextran sulfate sodium (DSS; MW=36000-50000, MP Biomedicals, LLC, Solon, OH, USA) in the drinking water at P90 *ad libitum* for 5 days followed by regular water for an additional 3-5 days. DSS of this molecular weight induces diffuse colitis from cecum to distal large bowel [23]. Susceptibly of DSS to induce colitis differs amongst different mouse strains [24,25]. Therefore, we used 3% DSS in our experiments with C57BL/6J based on our previous work [8,19], and 5% DSS [26] for the germ free mouse experiment. The animals were weighed daily and colonic length measurements were performed at the end of the experiments following CO_2_ asphyxiation. Weight loss (positively) and colonic length (negatively) correlates with the histological severity of colitis during these experiments. Therefore, we decided to follow weight loss and colon length as the primary clinical outcomes measuring colitis severity in our DSS experiments. Additionally, colons were longitudinally transected and processed for histology (described below).

### Fecal transfer into germ-free mice

To examine the effect of prenatal MD supplementation on postnatal microbiome function in respect to acute colitis we performed fecal microbiome transfer from the cross-fostered, prenatally MD supplemented animals (MD-C) and controls (C–C) into germ-free (GF) mice according to Vijay-Kumar et al. [27]. The donors consisted of 3 females and 2 males from each group which were not exposed to DSS at P90.Ten, 12 week-old, male Swiss-Webster (SW) GF mice were derived by cesarean section and maintained under GF conditions. Those were delivered by specific GF shipping (Taconic Farms Inc. Hudson, NY, USA). Cecal contents were suspended in phosphate buffered saline ([PBS] 2.5 ml per cecum) and pooled from the 5 mice in the MD-C or C-C groups following CO_2_ asphyxiation. The pooled content from the two groups was administered immediately after delivery via gavage into 5-5 randomly selected GF mice (0.1 ml per mouse). The transplanted mice were fed by control diet (NIH-31) for 5 days to allow for microbiome reconstitution and then exposed to 5% DSS to induce acute colitis. Body weight was measured daily. At the end of the experiment at day 8, the mice were euthanatized by CO2 asphyxiation. Colonic lengths were measured and histological severity of inflammation was determined as described above.

### Tissue collection

At the end of the feeding periods, mice were sacrificed by CO_2_ asphyxiation between 11:00 AM and 2:00 PM without any previous food restriction. The colons were placed on ice, and transected longitudinally. Cecal feces were flash frozen on dry ice and stored at -80^o^C until further analysis. The transected colons were cleansed from feces, washed with ice cold normal saline, and the tissue were preserved by the "Swiss roll" technique in 10% formalin for hematoxylin and eosin (H&E) staining for histological examination. For other analyses (gene expression and microbiome analysis) mucosal cleansing was followed by the collection of colonic mucosa with a microscope slide [28] (excluding the cecum). The mucosal scrapings were flash frozen on dry ice, and stored at -80^o^C as earlier described [8].

### Isolation and manipulation of nucleic acids

All procedures were performed on colonic mucosal scrapings. Total RNA was isolated with the RNeasy Mini Kit (74106, Qiagen, Valencia, CA, USA) and stored at −80°C. Reverse transcription was executed with the Taqman Reverse Transcription Kit (N808-0234, Applied Biosystems, Branchburg, NJ, USA).

### Quantitative analysis of gene expression

Quantitative real-time PCR (qRT-PCR) was performed using the following TaqMan gene expression assays: beta actin, as housekeeping gene: *B-actin* (Mm00607939_s1; Applied Biosystems, Foster City, CA) and peroxisome proliferator activated receptor alpha: *Ppara* (Mm 00440939_m1; Applied Biosystems, Foster City, CA). Reactions were performed in a 96-well assay format. All samples were plated in triplicates for both the *B-actin* and the *Ppara* expression measurements. The following thermal cycler protocol was used in the qRT-PCR reactions: 48 °C for 2 min and 95 °C for 10 min before the first cycle; 95 °C for 15 s, and 60 °C for 1 min, repeated 40 times. Reactions were processed using Bio-Rad iCycler Thermal Cycler instrument (#170-8720, Bio-Rad, Hercules, CA). Expression differences were determined by DataAssist Software v 3.0 program (Life technologies, Grand Island, NY, USA).

### DNA extraction for microbial studies

Mucosal scrapings and cecal feces were centrifuged at 14,000 rpm for 30 seconds and re-suspended in 500µl RLT buffer (Qiagen, Valencia, CA) (with β- mercaptoethanol). Sterile 5mm steel beads (Qiagen, Valencia, CA) and 500µl sterile 0.1mm glass beads (Scientific Industries, Inc., NY, USA) were added for complete bacterial lyses in a Qiagen TissueLyser (Qiagen, Valencia, CA), run at 30Hz for 5min. Samples were centrifuged briefly and 100µl of 100% ethanol was added to a 100µl aliquot of the sample supernatant. This mixture was added to a DNA spin column, and DNA recovery protocols were followed as instructed in the QIAamp DNA Mini Kit (Qiagen, Valencia, CA, USA) starting at step 5 of the Tissue Protocol. DNA was eluted and diluted to a final concentration of 20ng/µl.

### Massively parallel sequencing

Bacterial tag-encoded Ion Torrent PGM amplicon pyrosequencing was performed using fusion primers targeting the 515F-806R region of the bacterial *16S rRNA* gene (primers: 515F: 5’ GTGCCAGCMGCCGCGGTAA and 806R: 5’ GGACTACVSGGGTATCTAAT). A one-step fusion PCR using HotStar Qiagen PCR reagents was utilized. For each sample, 3 stochastic PCR replications were performed. The 3 replicates were pooled together equally and emulsions were performed using Ion Onetouch instrument (Life Technologies, USA). Sequencing was then performed on an Ion Torrent PGM instrument. The sequencing procedures were performed at MR DNA (Molecular Research) Shallowater, TX (www.MrDNAlab.com). Raw data was submitted to NCBI SRA submission SRA061527, STUDY SRP017164, SAMPLE SRS375287 ALIAS PRJNA179439.

### Bacterial diversity data analysis

All failed sequence reads, bar codes, primers, low quality sequence ends, sequences with >6bp homopolymers, sequences with degenerate base pair calls, and sequences shorter than 200bp were removed. Sequences were de-noised, de-replicated, clustered at 3% divergence; chimera checked using a custom pipeline developed by MR DNA (www.mrdnalab.com) that is based upon Usearch algorithms (Drive5.com). Sequences were also entered into Qiime for alpha and beta diversity analysis including 20 jackknife iterations (www.qiime.org). Of the original 1255171 sequences 111723 sequences with an average of 5586 per sample were utilized for analysis. Taxonomic analysis was performed using BLASTn against a database of high quality *16S rRNA* bacterial sequences derived and cross validated between NCBI, Greengenes and RDP. Using a. NET and C# analysis pipeline the resulting BLASTn outputs were compiled. Sequences with identity scores greater than 97% (<3% divergence) were resolved at the species level, between 95% and 97% at the genus level, between 90% and 95% at the family level, between 85% and 90% at the order level, between 80% and 85% at the class level, and below this to the phylum (77% and 80%). Statistical tests including Principle Component Analysis using Unifrac-based distances, ANOVA with Tukey-Kramer Post-hoc, and hierarchal clustering (Wards minimum variance clustering with Manhattan distances) to evaluate microbiome results were performed with NCSS 2007 (Kaysville, UT, USA).

### Statistical and bioinformatic analysis

For the bioinformatic analysis of the microbiome data, please see the paragraphs above. Unpaired two tailed t-test, Mann–Whitney U-tests, and Fischer exact test was utilized in the group comparisons where statistical significance was declared at p<0.05. Error bars represent standard error of the mean (SEM).

## Results

### Combined supplementation of methyl-donors increase offspring colitis

We have previously shown that maternal supplementation with MDs during pregnancy and lactation lead to increased colitis susceptibility in young adult mice [19]. Here we examined whether one micronutrient of the MDs, or all 4 are required to augment offspring acute (DSS). colitis when supplemented to dams. Offspring, maternally supplemented with combined MD, had increased mortality (weight loss greater than 25% necessitating euthanasia) compared to those with individual micronutrient supplementation, or control (p=0.019) (Figure S2). Interestingly, as opposed to our previous observations [19] males had more severe colitis. Of the 30 mice that had to be euthanized earlier secondary to weight loss, 80% were males. If examined separately, MD male offspring still had a significantly (p=0.039) increased mortality compared to control males, but females did not (p=0.206).

### Prenatal MD Supplementation Is Sufficient to Augments Offspring Colitis

Our earlier work demonstrated that prenatal and infantile MD supplementation augmented colitis in mice, but pediatric supplementation did not [19]. Here we set forth to determine the critical period of early development (*in utero* versus lactation) during which MD supplementation can increase colitis susceptibly in young adulthood. A cross-fostering experiment was conducted to answer our specific question. Total pups in each of the groups who received DSS included: C-C n=17; MD-MD = 26; MD-C n=17 and C-MD n=20.

**Figure 1:**
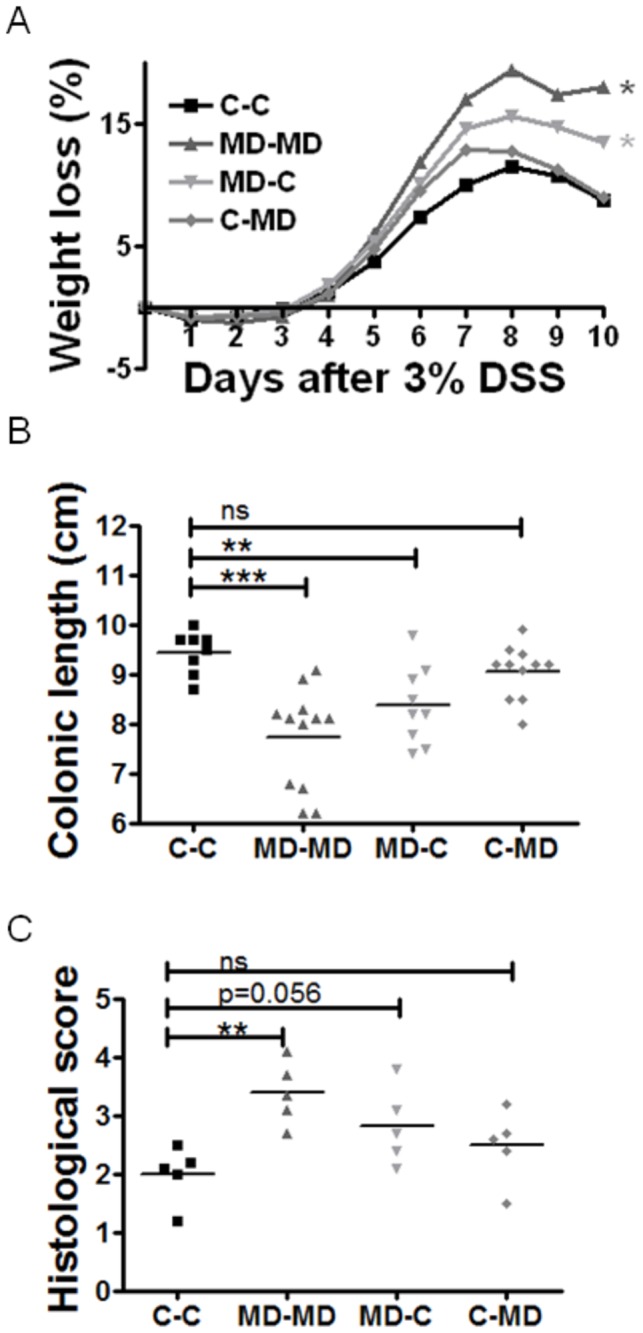
Prenatal MD supplementation is sufficient to worsen young adult male colitis. Dams were provided either a control (C) or methyl-donor (MD) diet before, during and following (lactation) pregnancy. Each litter was crossed fostered. Groups were designated as following: MD-MD if they had received MD diet *in utero* and lactation; C-C for control diet *in utero* and lactation; MD-C for MD *in utero* and control in lactation and the last group were C-MD that received control *in utero* and MD in lactation. Offspring were transferred to control diet at weaning (P21) and tested for susceptibility to colitis by 3% DSS exposure for 5 days in drinking water at P90. (A) The MD-MD and MD-C male mice showed significantly (*p<0.05) increased weight loss compared to the C-C. (B) Colonic lengths were significantly shorter (*p<0.05) in the MD-MD and MD-C compared to the negative control C-C. N=8-12 per group. (C) Histological severity of inflammation was consistent with the MD-MD group suffering a more severe colitis than control. There was a trend approaching significance for microscopically increased colitis in the MD-C group as well. N=5.

A gender specific colitis phenotype was repeatedly observed during the cross fostering experiments with males showing diet dependent colitis severity changes, while females did not. Only the MD-MD (positive control) and the MD-C males had significantly increased weight loss, decreased colonic length, and increased histological severity of colitis compared to the C-C male offspring. C-MD males did not demonstrate a significant difference compared to controls in all there mentioned parameters (weight loss p= 0.88; colonic length p=0.11 and histological severity p=0.21) (Figure 1).

### Prenatal MD supplementation is sufficient to increase colonic mucosal Pparα expression in offspring

**Figure 2:**
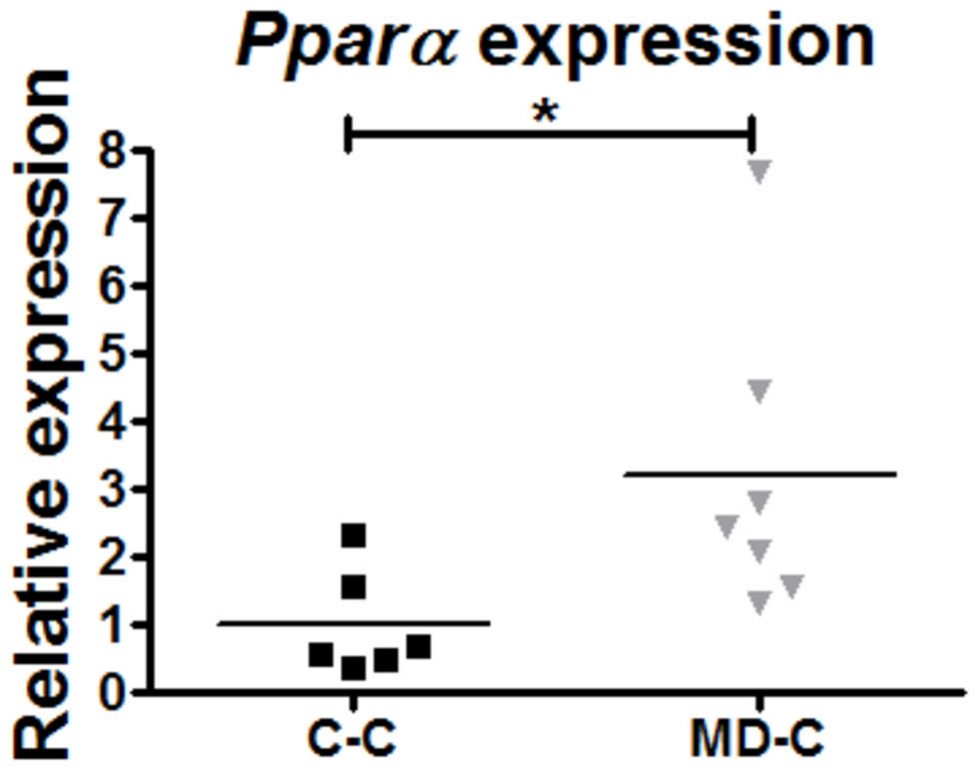
Increased expression of *Ppara* in prenatally MD supplemented young adult mice. *Ppara* expression by real-time RT-PCR measurements from colonic mucosal scrapings showed increased expression in the MD-C animals compared to C-C at P90 (U test, p=0.022; n=6-7).

The cross fostering experiment showed that prenatal MD supplementation is primarily responsible for the MD induced increase in colitis susceptibility, as opposed to its selective neonatal (lactation) supplementation. Our previous studies have demonstrated that maternally MD supplemented pups displayed DNA methylation changes at mucosal peroxisome proliferator-activated receptor alpha (Ppara), where expression inversely correlated with this epigenetic modification [19]. Since *Ppara* has been recognized as a modifier of murine colitis [29,30] we examined as an example whether prenatal MD supplementation is sufficient to persistently change its colonic mucosal expression. *Ppara* expression was significantly increased in the young adult MD-C (n=7) group (p=0.022) as compared to C-C (n=6) (Figure 2).

### Prenatal MD supplementation induced mucosal and fecal microbiome separation in young adult mice

**Figure 3:**
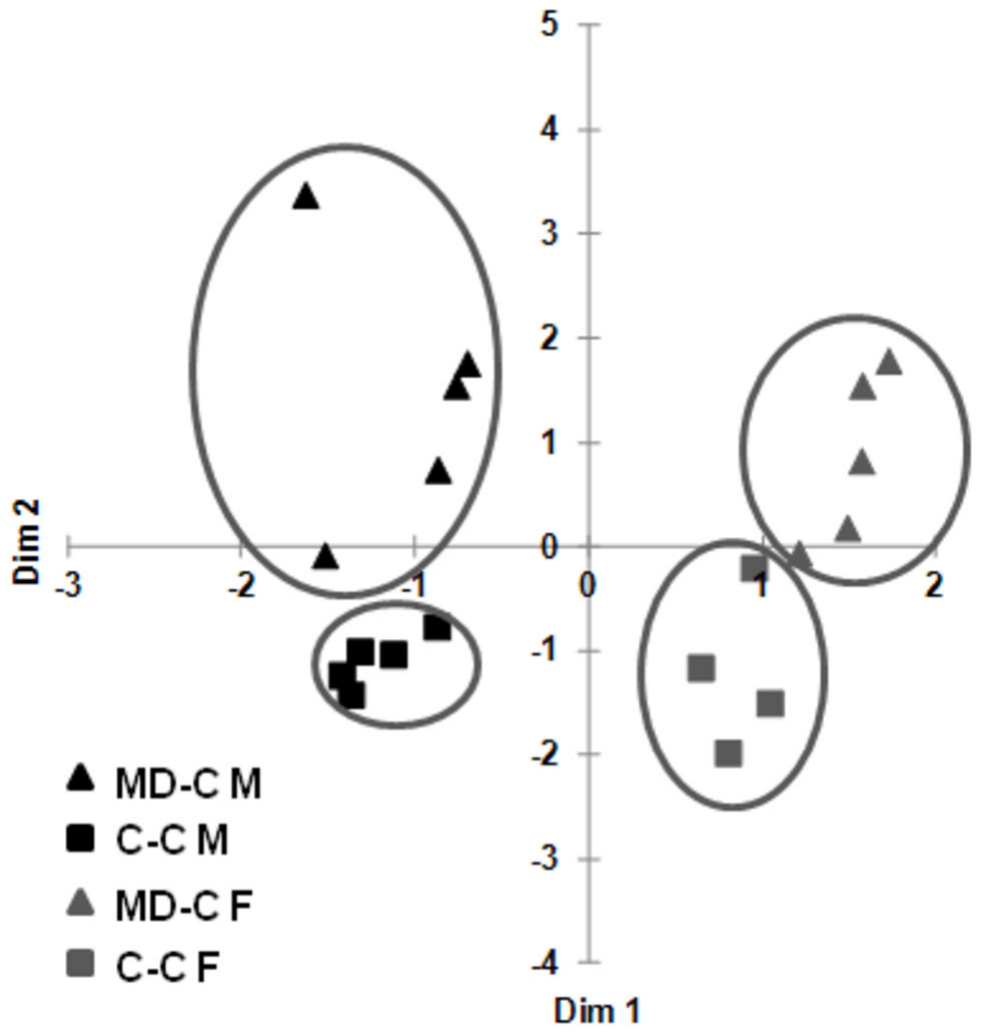
Fecal (F) and colonic mucosal (M) microbiome separation in prenatally MD supplemented (MD-C) young adult mice. Cecal content (feces) and colonic mucosal scrapings from non-DSS exposed C-C and MD-C animals were processed for microbiome analysis at P90. Principal coordinates analysis (PCoA) of weighted UniFrac measures showed separation of fecal and mucosal microbiomes in the prenatally MD supplemented (MD-C) mice compared to the control cross fostered group (C–C). N=5 per group.

The gut microbiome is an anticipated factor in IBD pathogenesis [31,32] and dietary influences can modify its composition [22]. In the meantime, little is known about prenatal environmental/dietary effects (i.e. during a sterile stage of development) transmitting microbiome composition changes into postnatal development. Our previous investigations indicated that MD supplementation may modify host maturation towards maintaining a colitogenic microbiome independently from the inherited dam microbiomes [33]. To confirm these findings, we examined the effects of prenatal MD supplementation on colonic mucosal and luminal (cecal content) microbiomes in the MD-C and C-C groups. Principal coordinates analysis (PCoA) showed a prenatal MD supplementation dependent separation both in mucosal and fecal (from cecum) microbiomes (Figure 3). The PCoA also indicated that the mucosal microbiome separated more (greater relative distance) from the luminal in the MD-C group than in the C-C group.

### Prenatal MD supplementation induced young adult mucosal microbiome changes

At the phyla level, prenatal MD supplementation associated with an increase in Firmicutes (p=0.03) and *Cyanobacteria* (p=0.04) and a decrease in Verrucomicrobia (p=0.02) compared to controls (C–C) in the colonic mucosa (Table 1).

**Table 1:** The effects of prenatal maternal diets on colonic mucosa associated bacterial taxa.

**Mucosa C-C and MD-C**	**C-C**	**MD-C**		
**Phyla**	**%**	**%**	**P**	**U**
Verrucomicrobia	0.01	0	0.0185	NA
Firmicutes	45.19	60.29	0.0304	0.0079
*Cyanobacteria*	0	0.02	0.0431	NA
**Genera**	**%**	**%**	**P**	**U**
*Anaerostipes*	0.20	1.48	0.0023	0.0079
*Akkermansia*	0.01	0	0.0185	NA
*Allobaculum*	35.16	17.78	0.0222	0.0159
*Lactococcus*	0	0.02	0.0461	NA
**Species**	**%**	**%**	**P**	**U**
*Clostridium* *sordellii*	0.27	0	0.0018	NA
*Anaerostipes caccae*	0.20	1.48	0.0023	0.0079
*Akkermansia* *muciniphila*	0.01	0	0.0185	NA
*Roseburia* *eubacterium* *rectale*	0.13	0.60	0.0223	0.0079
*Lactococcus lactis*	0	0.02	0.0461	NA

Significant bacterial abundance differences at the phyla, genera and species level between the mucosal MD-C and C-C groups (MD-C: MD *in utero* and control diet in lactation, C: control diet *in utero* and lactation). P and U values represent probability of non-paired, two tailed T test and the non-parametric Mann-Whitney test.

**Figure 4:**
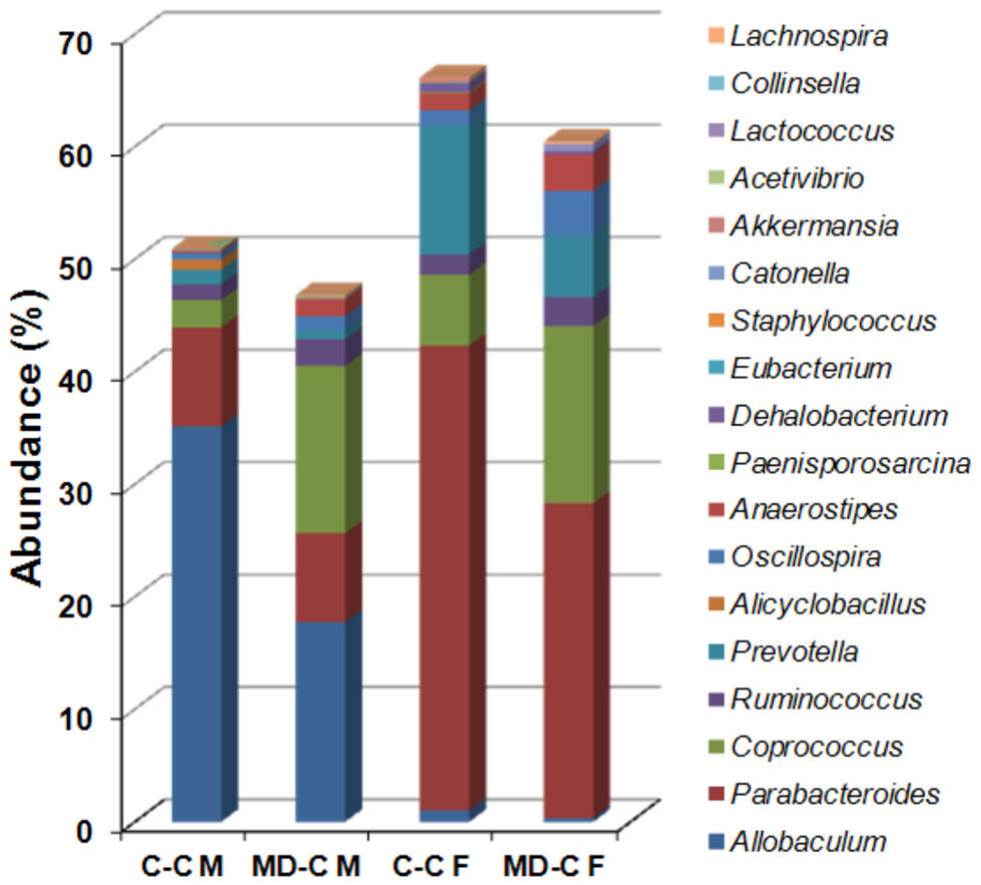
Genera level separation of colonic mucosal and fecal microbiomes upon prenatal MD supplementation. Combined representation of the 10-10 most prominently (by T test p value) differing genera in the mucosal and fecal (cecal content) samples, respectively, secondary to prenatal MD supplementation. *Anaerosptipes* and *Akkermansia* overlapped between the mucosal and fecal comparisons. Hence 18 genera are depicted. Sixteen (90%) out of the 18 genera changed similarly in abundance between mucosa (M) and feces (F) upon prenatal MD supplementation at P90. Prenatally MD supplemented (MD-C) and control cross fostered groups (C–C) are compared. N=5.

At the genera level, *Anaerostipes* and *Lactococcus* were increased in the MD-C mucosa (p<0.05), while *Allobaculum* and *Akkermansia* decreased compared to the C-C mucosa. *Allobaculum* was the most abundant genera to be affected by prenatal MD supplementation in the colonic mucosa (17.8% MD-C; 35.2% in C-C; p=0.02) (Figure 4 and Table 1).

There was an increase at the species level in *Anaerostipes caccae,* *Roseburia* *eubacterium* *rectale* and *Lactococcus lactis*, whereas a decrease in *Clostridium* *sordelli* and *Akkermansia* *mucinphilia* occurred secondary to the prenatal MD supplementation (Table 1).

### Prenatal MD supplementation induced fecal microbiome changes

At the phyla level, prenatal MD supplementation resulted in an increased abundance of Firmicutes (p=0.02) and decreased Bacteroidetes (p=0.03) within cecal content (feces) microbiomes compared to controls (Table 2).

**Table 2:** The effects of prenatal maternal diets on feces associated bacterial taxa.

**Feces C-C and MD-C**	**C-C**	**MD-C**		
**Phyla**	**%**	**%**	**P**	**U**
Firmicutes	42.54	64.38	0.0248	0.0317
Bacteroidetes	55.38	33.91	0.0259	0.0556
**Genera**	**%**	**%**	**P**	**U**
*Lachnospira*	0.02	0.18	0.0017	0.0079
*Oscillospira*	1.30	3.95	0.0045	0.0159
*Ruminococcus*	1.79	2.55	0.012	0.0079
*Parabacteroides*	41.29	27.97	0.0259	0.0159
*Eubacterium*	0.09	0.01	0.0292	0.0317
*Catonella*	0.02	0.08	0.0304	0.0079
**Species**	**%**	**%**	**P**	**U**
*Lachnospira* *pectinoschiza*	0.02	0.18	0.0017	0.0079
*Oscillospira* *guilliermondii*	1.30	3.95	0.0045	0.0159
*Roseburia* *eubacterium* *rectale*	0.53	1.41	0.009	0.0079
*Parabacteroides* *distasonis*	41.29	27.97	0.0259	0.0159
*Eubacterium* *siraeum*	0.09	0.01	0.0292	0.0317
*Catonella* * (species unknown)*	0.02	0.08	0.0304	0.0079

Significant bacterial abundance differences at the phyla, genera and species level between the fecal samples in the MD and C groups (MD-C: MD *in utero* and control diet during lactation, C: control diet *in utero* and during lactation). P and U values represent probability of non-paired, two tailed T test and the non-parametric Mann-Whitney test.

At the genera level, the MD-C group had and increase in *Lachnospira* *,* *Oscillospira* *,* *Ruminococcus* *and* *Catonella* (all Firmicutes). *Parabacteroides* and *Eubacterium* were decreased in the MD-C group. *Parabacteroides* was the most abundant genera to be affected by prenatal MD supplementation in feces (27.97% MD-C; 41.29% in C-C; p=0.026) (Figure 4 and Table 2).

At the species level the prenatally MD supplemented young adult mice had increased *Lachnospira* *pectinoschiza* *,* *Oscillospira* *guilliermondii* , *Roseburia* *eubacterium* *rectale* and *Catonella* (species unknown)*.* *Parabacteroides* *distasonis* *and* *Eubacterium* *siraeum* were higher in the C-C (Table 2).

### Prenatal MD supplementation induced increased mucosal-luminal microbiome partitioning

Fecal microbiomes differ from mucosa associated microbial communities [34]. The mucosal microbiome is possibly more relevant for intestinal immunomodulation [35]. Here we examined if prenatal MD supplementation affected mucosa to feces partitioning in young adult mice. The PCoA analysis performed on the mucosal and fecal microbiomes indicated that prenatal MD supplementation increased the relative difference between the mucosal and fecal microbiomes (Figure 4). Consistent with this observation there were more taxa at the genera and species level with significant difference between fecal and mucosal microbiomes secondary to the prenatal nutritional intervention employed (Tables S1 and S2). While there were 12 genera significantly different between cecal feces and colonic mucosa in the control group (out of 77 detected), this number increased to 22 in the MD-C group (out of 81 detected) (p=0.084). The abundance of 20 species was different between feces and mucosa in the C-C group (out of 128 detected), and 33 species in the MD-C group (out of 127 detected) (p=0.046).

### Prenatal MD supplementation induced the nurturing of a colitogenic microbiome

**Figure 5:**
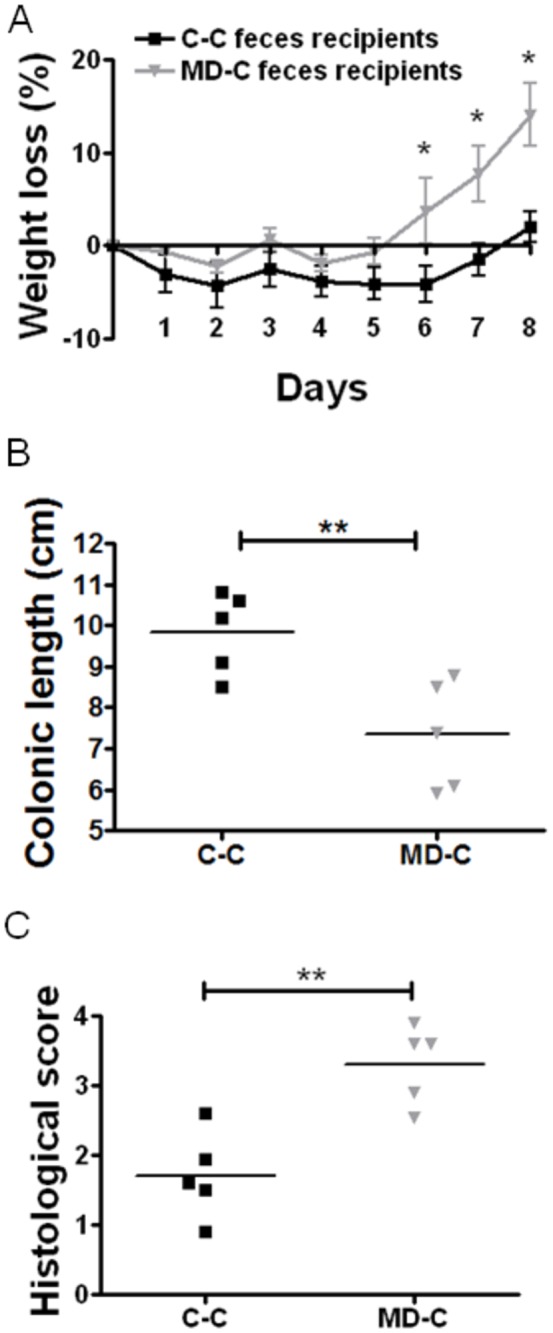
Significantly augmented colitis in germ free recipients of MD-C feces. 5-5 germ free male mice were gavaged with pooled cecal content (feces) from either prenatally MD supplemented animals (MD-C), or controls (C–C). After 5 days the animals were tested for colitis susceptibility by 5% DSS exposure in drinking water for 5 days. At day 8, the mice were euthanatized. Colonic length was measured and colonic tissue was examined histologically. (A) Weight loss upon 5% DSS challenge was significantly greater in the recipients of MD-C feces at days 6, 7, and 8 (*p<0.05). (B) The increased severity of colitis was also reflected by significant colonic length shortening in the recipients of MD-C feces (p=0.0099) compared to control. (C) A greater degree of tissue damage and inflammation (p=0.0028) was observed in the MD-C fecal recipients compared to the recipients of control stool. N=5.

The metagenomic analyses showed that prenatal MD supplementation does modify large intestinal microbiome composition in young adult mice. We wished to examine the functional consequences of this nutritionally induced microbiome variation in respect to colitis. Therefore, we performed cecal content transfer into GF mice from 5 C–C and 5 MD-C animals and tested the recipient’s susceptibility to DSS colitis. The GF mice transplanted with feces from the MD-C group had worse colitis (greater loss of body weight, shorter colonic length (p<0.01) and higher histological scores (p=0.0028), indicating increased severity of colitis) as compared to the recipients of C-C feces (Figure 5).

## Discussion

The importance of microbial composition changes during infancy has been observed in mammalian models in respect to mucosal immune responses [36] and colitis susceptibility [37]. The oral and gut mucosa can react to microbial changes by epigenetic modification at select genes relevant for intestinal immunomodulation such as *TLR4* [38], *hBD2* (the gene of the antimicrobial peptide human β-defensin 2), CC chemokine ligand 20 (*CCL20*), and others [39]. Such epigenetic modification may be dynamic and dependent on the presence of specific microbes [40]. Very recently, infantile exposure to specific pathogen free microbiome in germ free mice has been shown to decrease the methylation, hydroxymethylation and gene expression of *Cxcl16* (the ligand of chemokine receptor Cxcr6) in the colon, whereas later (at 5 weeks of age, i.e. late pediatric development in mice) exposure to the microbiome did not [41]. Cxcl16 was critical for the accumulation of invariant natural killer cells into the tissues and for sensitization to oxazolone induced colitis. This work provided the first evidence for microbial influences to induce epigenetic changes relevant for mammalian colitis during a specific period of postnatal development. Conversely, the potential for fetal nutritional developmental programming of the host to modulate microbial composition in respect to disease susceptibility has not been examined before.

Here we found that one specific MD micronutrient was insufficient for modifying colitis when maternally supplemented to offspring. On one hand, this result may be relevant for human maternal supplementation with prenatal vitamins, indicating that the combined provision of those should be reconsidered in light of the emergence of IBD in the developed world. On the other hand, the results are reassuring in respect to the select supplementation of one micronutrient, such as folic acid. Examining the extraordinarily high supplementation of a single micronutrient (which is not the case in current obstetric practice) to account for the combined 4 MD supplementation in a dose equivalent manner seemed clinically irrelevant for us. Therefore, such experiments were not performed.

We further examined the critical developmental period for maternal micronutrients to modify offspring colitis susceptibility in young adulthood. The cross-fostering experiments conclusively showed that prenatal MD supplementation is more important in modulating postnatal susceptibility to colitis than the early postnatal (lactation) period, at least in male mice. Perinatal stress upon cross-fostering may have contributed to the gender specific nature of MD supplementation in these experiments as opposed to our previous results without cross-fostering [19]. Such gender specific epigenetic changes secondary to neonatal stress have certainly been observed in mice [42]. In addition, male mice have an increased DSS colitis susceptibly compared to the female gender [24], which may have also contributed to our findings.

Regardless of the gender specific nature of augmented DSS colitis susceptibility following prenatal MD supplementation, we found that the nutritional intervention increased colonic mucosal *Ppara* expression up to P90 in the offspring. This finding coincided with maternal MD supplementation inducing persistently decreased methylation and increased expression of *Ppara* in the colonic mucosa of the offspring [19]. According to our results here, prenatal MD supplementation is sufficient to produce this somatic modification. Interestingly, other maternal dietary measures, such as protein-restriction have been shown to decrease promoter methylation [43] and increase the expression of hepatic *Ppara* in rats [44], indicating that the epigenetic regulation of this nuclear receptor is sensitive to prenatal nutrition. While there is limited information on *Ppara* in respect to colitis modulation, agonistic stimulation of the molecule with fenofibrate [45] and the less selective Wy-14,643 [46] support its protective role in chronic and acute murine models of IBD. Therefore, our findings show that selectively prenatal micronutrient supplementation is sufficient to modify the host immune-enterocyte network.

Importantly, the gut is normally sterile at birth, and colonization begins at delivery [47]. This colonization is influenced by the mode of delivery [48], breast or formula feeding [49], and gestational age [50]. As emphasized above, the pattern of infantile microbial colonization can impact mucosal immune responses [36] and susceptibility to immunologically mediated disorders (such as asthma and colitis) [37,41] in animal models. These findings are relevant for human pathology since prebiotic and probiotic supplementation (i.e. early postnatal microbiome modulation) in infancy has a protective role in atopic conditions [51] and necrotizing enterocolitis [52], for example. Similarly, antibiotic exposure, especially before 1 year of age, associates with an increased incidence of IBD [53]. In the meantime, while the influence of prenatal environmental exposures on offspring susceptibility to chronic inflammatory conditions has been emphasized [54], direct studies are scarce in this respect. One reason for the lack of conclusive findings about prenatal environmental influences impacting postnatal phenotypes is the difficulty of discriminating prenatal exposures from neonatal/early postnatal ones in maternal supplementation experiments. This challenge can be overcome by neonatal cross-fostering. This was the reason for us pursuing cross-fostering of murine offspring following maternal MD supplementation to dams on control diet and vice-versa.

Our earlier investigations indicated that the maternal MD supplementation induced prolonged microbiome variation is independent from the microbiome of the dams (i.e. independent from perinatal colonization) [33]. The findings of this work conclusively show that prenatal MD supplementation is sufficient to modulate gut microbiome composition independently from postnatal maternal exposures. Importantly, the fecal transfer experiments into germ free mice revealed that the prenatal MD supplementation induced a colitis prone microbiome in the offspring. The establishment of this colitis prone microbiome was likely secondary to a complex dysbiosis that manifested as a consequence of prenatal MD dependent changes in host biological structures relevant for intestinal immunomodulation (such as *Ppara*, for example). Current evidence suggests that it is intestinal dysbiosis that is important for IBD pathogenesis rather than abundance variation in a single taxon [32]. Nevertheless, we compared the changes in select taxa to the available literature on mammalian colitis and microbiome variation. The genera *Allobaculum* decreased in abundance both in the mucosal and fecal microbiomes secondary to the prenatal MD supplementation. This taxon constitutes of short-chain fatty acid (SCFA) producing bacteria [55]. SCFAs are important anti-inflammatory mediators in the gut [56]. Therefore, the decreased abundance of *Allobaculum* may have contributed to the colitis prone nature of the MD-C microbiomes. Similarly, *Parabacteroides* including *P* *. distasonis* decreased in the MD-C microbiomes. Members of this genus were less abundant in patients with ulcerative colitis (UC) and irritable bowel syndrome [57], and oral administration of *P* *. distasonis* decreased the severity of DSS colitis in mice [58]. Furthermore, although low in abundance even in control microbiomes, *Akkermansia* , and *A* *. municiphila* specifically further decreased in the prenatally MD supplemented offspring, while *Ruminococcus* increased. Similar changes in *A* *. municiphila* and *Ruminococci* were detected in both UC and Crohn disease patients [59]. We have recently found decreased abundance of *Eubacterium* in the colonic mucosa of treatment naïve pediatric CD patients [60]. MD-C microbiomes also had decreased *Eubacteria*. These taxa changes indicate that the prenatal MD supplementation evoked microbiome variation in mice resembles either colitis prone or a colitis induced mammalian (including human) microbiome. However, opposing changes between our metagenomic results and human IBD were detected as well. One example for this is *Prevotella* , where a decrease was observed in the MD-C (colitis prone) mice compared to control, while this genera was increased in patients with IBD [61,62]. These observations repeatedly suggest that an overall dysbiosis (disproportionate changes between colitogenic and anti-inflammatory bacteria) within the MD-C microbiomes is responsible for the colitis prone phenotype following prenatal MD supplementation.

This work includes the first investigation of selective exposure to increased amounts of epigenetically active micronutrients during prenatal development. The dietary intervention was found to evoke prolonged colonic mucosal modification in respect to *Ppara* (a nuclear receptor relevant for intestinal immunomodulation) expression, as an example. Although the nutritional exposure was provided during the sterile development of the intestines, it induced the postnatal nurturing of a colitis prone (colitogenic) microbiome in the animals. Therefore, our findings support the prenatal nutritional origins paradigm of IBD, where early metabolic exposures can imprint the mammalian host to harbor an inflammation prone microbiome at young adulthood, when the incidence of the disorders peak. Further efforts will be required to elucidate the intricate modulation of the host immune-enterocyte network and commensal microbiota communication that occurs upon prenatal micronutrient exposures. Such work will promote our better understanding of the developmental origins of IBD.

## Supporting Information

Figure S1 **Schematic description of the cross-fostering experiment employed.** Dams were provided either a control (C) or methyl-donor (MD) diet before, during and following (lactation) pregnancy. Each litter was crossed fostered. Groups were designated as following: MD-MD if they had received MD diet *in utero* and lactation; C-C for control diet *in utero* and lactation; MD-C for MD *in utero* and control in lactation and the last group were C-MD that received control *in utero* and MD in lactation. Offspring were transferred to control diet at weaning (P21) and tested for susceptibility to colitis by 3% DSS exposure for 5 days in drinking water at P90.
(TIF)

Figure S2 **Mortality (>25% weight loss) following dextran sulfate sodium (DSS) exposure.** Dams were provided combined, or select supplementation of methyl-donors (MD: betaine, folic acid [FA], vitamin B12 [B12], and choline) 2 weeks before, during, and following (lactation) pregnancy. Offspring were transferred to control diet at weaning (P21) and tested for susceptibility to colitis by 3% DSS exposure for 5 days in drinking water at P90. Only the combined supplementation of MDs worsened colitis severity (i.e. increased number of animals with >25% weight loss necessitating euthanasia = “mortality”) significantly (Fischer exact p=0.019) compared to control. Control n= 20, MD n= 20, betaine n=19, folic acid n=25 and choline n= 16.
(TIF)

Table S1 **The effects of prenatal control diets on colonic mucosa and feces associated bacterial taxa.**
(PDF)

Table S2 **The effects of prenatal MD supplemented diet on colonic mucosa and feces associated bacterial taxa.**
(PDF)
