# Supplementary figures and images for: Prenatal Methyl-Donor Supplementation Augments Colitis in Young Adult Mice
Source: PLoS One. 2013 Aug 19;8(8):e73162. doi: 10.1371/journal.pone.0073162 (PMC3747105; doi:10.1371/journal.pone.0073162)

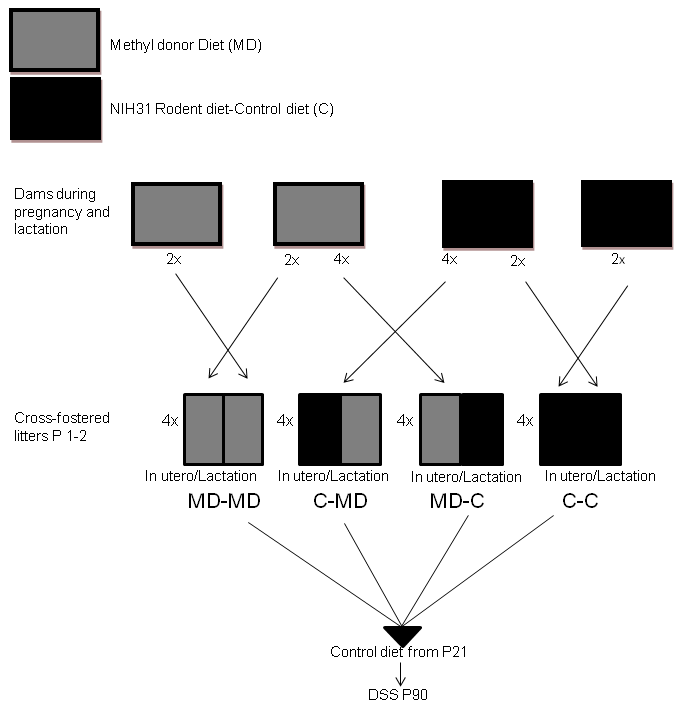

Supplement: Figure S1 — Schematic description of the cross-fostering experiment employed. Dams were provided either a control (C) or methyl-donor (MD) diet before, during and following (lactation) pregnancy. Each litter was crossed fostered. Groups were designated as following: MD-MD if they had received MD diet in utero and lactation; C-C for control diet in utero and lactation; MD-C for MD in utero and control in lactation and the last group were C-MD that received control in utero and MD in lactation. Offspring were transferred to control diet at weaning (P21) and tested for susceptibility to colitis by 3% DSS exposure for 5 days in drinking water at P90. (TIF) [file pone.0073162.s001.tif]

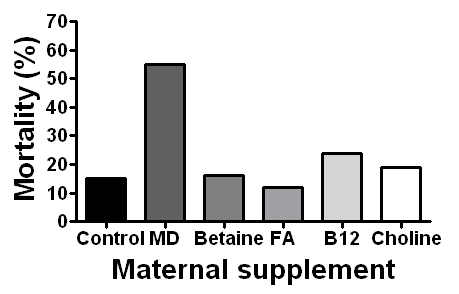

Supplement: Figure S2 — Mortality (>25% weight loss) following dextran sulfate sodium (DSS) exposure. Dams were provided combined, or select supplementation of methyl-donors (MD: betaine, folic acid [FA], vitamin B12 [B12], and choline) 2 weeks before, during, and following (lactation) pregnancy. Offspring were transferred to control diet at weaning (P21) and tested for susceptibility to colitis by 3% DSS exposure for 5 days in drinking water at P90. Only the combined supplementation of MDs worsened colitis severity (i.e. increased number of animals with >25% weight loss necessitating euthanasia = “mortality”) significantly (Fischer exact p=0.019) compared to control. Control n= 20, MD n= 20, betaine n=19, folic acid n=25 and choline n= 16. (TIF) [file pone.0073162.s002.tif]
