# Supplementary material for: Prenatal Methyl-Donor Supplementation Augments Colitis in Young Adult Mice
Source: PLoS One. 2013 Aug 19;8(8):e73162. doi: 10.1371/journal.pone.0073162 (PMC3747105; doi:10.1371/journal.pone.0073162)
Supplement: Table S1 — The effects of prenatal control diets on colonic mucosa and feces associated bacterial taxa. (PDF) [file pone.0073162.s003.pdf]

Table S1. The effects of prenatal control diets on colonic mucosa and feces associated bacterial taxa.

	Mucosa	Feces	
**Phyla**	**%**	**%**	**Paired T test**
Tenericutes	43.87	1.51	0.0000001
Bacteroidetes	10.40	55.38	0.0002
Verrucomicrobia	0.01	0.27	0.0458
**Genera**	**%**	**%**	**Paired T test**
Allobaculum	35.16	1.04	0.001
Mogibacterium	0.02	0.08	0.004
Parabacteroides	8.81	41.29	0.0043
Rfn20	0.01	0.03	0.0096
Prevotella	1.29	11.52	0.0124
Turicibacter	3.44	0.45	0.0204
Brevibacillus	13.93	0.54	0.0242
Sphingobium	0.05	0	0.0288
Dehalobacterium	0.04	0.81	0.0299
Lactobacillus	5.46	0.48	0.0327
Blautia	4.29	13.69	0.0336
Shimazuella	0.04	0.00	0.0466
**Species**	**%**	**%**	**Paired T test**
Mogibacterium (species unknown)	0.02	0.08	0.004
Parabacteroides distasonis	8.81	41.29	0.0043
Clostridium sordellii	0.27	0.02	0.0063
Rfn20 (species unknown)	0.01	0.03	0.0096
Prevotella falsenii	0.28	2.33	0.017
Prevotella copri	0.26	1.90	0.0184
Turicibacter (species unknown)	3.44	0.45	0.0204
Lactobacillus reuteri	0.30	0	0.0214
Clostridium celerecrescens	0.24	1.20	0.0227
Roseburia eubacterium rectale	0.13	0.53	0.023
Ruminococcus flavefaciens	0.08	0.29	0.0235
Brevibacillus laterosporus	13.93	0.54	0.0242
Allobaculum stercoricanis	19.27	0.99	0.0275
Sphingobium yanoikuyae	0.05	0.00	0.0288
Dehalobacterium (species unknown)	0.04	0.81	0.0299
Blautia producta	4.27	13.56	0.0364
Lactobacillus iners	5.13	0.43	0.0404
Prevotella baroniae	0.05	0.30	0.0435
Shimazuella (species unknown)	0.04	0	0.0466
Lactobacillus intestinalis	0.03	0	0.0476

Significant bacterial abundance differences at the phyla, genera, and species level between the mucosal and fecal samples of the C-C group (C: control diet *in utero* and lactation). Paired T test comparisons between the two groups (see Materials and Methods). The most prominently

differing phyla between mucosal and feces was Tenerictues. A total of 12 genera and 20 species were significantly different in the mucosa as compared to the luminal content in the control group.
