# Supplementary material for: Prenatal Methyl-Donor Supplementation Augments Colitis in Young Adult Mice
Source: PLoS One. 2013 Aug 19;8(8):e73162. doi: 10.1371/journal.pone.0073162 (PMC3747105; doi:10.1371/journal.pone.0073162)
Supplement: Table S2 — The effects of prenatal MD supplemented diet on colonic mucosa and feces associated bacterial taxa. (PDF) [file pone.0073162.s004.pdf]

Table S2.The effects of prenatal MD supplemented diet on colonic mucosa and feces associated bacterial taxa.

**MD-C Mucosa-Feces**	**Mucosa**	**Feces**	
Phyla	**%**	**%**	**Paired T test**
Bacteroidetes	8.80	33.91	0.000012
Tenericutes	29.84	1.17	0.0026
Cyanobacteria	0.02	0	0.0216
**Genera**	**%**	**%**	**Paired T test**
Pseudobutyrivibrio	0.07	0.12	0.0001
Parabacteroides	7.88	27.97	0.0039
Butyrivibrio	0.15	0.49	0.0041
Psb	0.07	0.22	0.0112
Lachnospira	0.01	0.18	0.0126
Brevibacillus	13.64	0.29	0.0179
Allobaculum	17.78	0.34	0.018
Caloramator	0.03	0.15	0.0192
Granulicatella	9.80	0.15	0.0202
Anaerostipes	1.48	3.25	0.0218
P	1.57	0.06	0.0222
Prevotella	0.78	5.52	0.0223
Blautia	3.92	21.61	0.0225
Dorea	0.06	0.01	0.0231
Enterococcus	0.07	0	0.0252
Pontibacter	0.06	0.19	0.0269
Alkaliphilus	0.03	0.28	0.0287
Tannerella	0.03	0.19	0.0293
Roseburia	2.02	4.43	0.0318
Clostridium	13.92	6.01	0.0329
Oscillospira	1.24	3.95	0.0342
Shimazuella	0.03	0.01	0.0457
**Species**	**%**	**%**	**Paired T test**
Pseudobutyrivibrio ruminis	0.07	0.12	0.0001
Parabacteroides distasonis	7.88	27.97	0.0039
Clostridium orbiscindens	0.26	0.72	0.0046
Butyrivibrio hungatei	0.05	0.35	0.0061
Clostridium methylpentosum	0.06	0.22	0.01
Psb	0.07	0.22	0.0112
Blautia ruminococcus obeum	0.13	0.32	0.0122
Lachnospira pectinoschiza	0.01	0.18	0.0126
Brevibacillus laterosporus	13.64	0.29	0.0179
Allobaculum stercoricanis	17.70	0.33	0.018
Clostridium cocleatum	10.37	0.40	0.0188
Caloramator (species unknown)	0.03	0.15	0.0192
Granulicatella adiacens	9.75	0.15	0.0202
Clostridium perfringens	0.28	0.01	0.0208
Anaerostipes caccae	1.48	3.25	0.0218
P	1.57	0.06	0.0222

Species continued	%	%	Paired T test
Dorea formicigenerans	0.06	0.01	0.0231
Blautia producta	3.79	21.28	0.0238
Clostridium celerecrescens	0.43	1.06	0.0251
Enterococcus faecalis	0.07	0	0.0252
Prevotella copri	0.20	1.03	0.0264
Prevotella multiformis	0.11	0.30	0.0264
Pontibacter korlensis	0.06	0.19	0.0269
Granulicatella elegans	0.04	0.00	0.0275
Tannerella forsythia	0.03	0.19	0.0293
Alkaliphilus transvaalensis	0.02	0.28	0.0302
Prevotella falsenii	0.26	1.86	0.0307
Oscillospira guilliermondii	1.24	3.95	0.0342
Roseburia eubacterium rectale	0.60	1.41	0.0351
Allobaculum sp id4	0.08	0.02	0.0386
Roseburia faecis	1.43	3.02	0.0387
Shimazuella (species unknown)	0.03	0.01	0.0457
Prevotella intermedia	0.11	1.12	0.0486

Significant bacterial abundance differences at the phyla, genera, and species level between the mucosal and fecal samples of the MD-C Group (MD-C: MD *in utero and* control diet in lactation). Paired T test comparisons between the two groups (see Materials and Methods). A total of 22 genera and 33 species were significantly different between mucosa and feces in the prenatally MD supplemented group.
